# Dual‐antibiotic bone cement (gentamicin + vancomycin) in preventing and treating infections during revision knee arthroplasty in high‐risk patients

**DOI:** 10.1002/jeo2.70638

**Published:** 2026-01-27

**Authors:** Shengdong Yang, Nicolas Cance, Cécile Batailler, Tristan Ferry, Pierre Longlune, Luca Andriollo, Hannes Vermue, Sébastien Lustig

**Affiliations:** ^1^ Orthopaedics Surgery and Sports Medicine Department IFA Medical Center of Excellence, Croix Rousse Hospital, Hospices Civils de Lyon Lyon North University Hospital Lyon France; ^2^ IFSTTAR, LBMC UMR_T9406, Claude Bernard Lyon 1 University University of Lyon Lyon France; ^3^ Centre interrégional de Référence pour la prise en charge des Infections Ostéo‐Articulaires complexes (CRIOAc Lyon), Hospices Civils de Lyon Lyon France; ^4^ Service de Maladies Infectieuses et Tropicales, Hôpital de la Croix‐Rousse, Hospices Civils de Lyon Lyon France; ^5^ Ortopedia e Traumatologia, Fondazione Poliambulanza Istituto Ospedaliero Brescia Italy; ^6^ Artificial Intelligence Center Alma Mater Europaea University Vienna Austria; ^7^ Ortopedia e Traumatologia Università Cattolica del Sacro Cuore Roma Italy; ^8^ Department of Orthopedic Surgery and Traumatology Ghent University Hospital Ghent Belgium

**Keywords:** dual‐antibiotic bone cement, gentamicin, knee revision arthroplasty, periprosthetic joint infection, vancomycin

## Abstract

**Purpose:**

This study aimed to evaluate the treatment and preventive effects, as well as the safety, of dual‐antibiotic bone cement (DABC; gentamicin + vancomycin) in revision total knee arthroplasty (RTKA) in high‐risk patients.

**Methods:**

This retrospective observational study included patients who underwent RTKA for septic or aseptic indications with intraoperative application of DABC (Copal G + V, Heraeus‐Medical GmbH) at our centre between December 2015 and December 2022. Patients were followed for a minimum of 2 years. Postoperative infection rates were documented, and preoperative patient risk was calculated using the periprosthetic joint infection (PJI) risk calculator. Microbiological profiles and antibiotic resistance patterns of postoperative infections were analysed, and all complications were recorded.

**Results:**

A total of 85 patients were included. The overall postoperative infection rate after DABC use was 17.6% (15/85), 95% confidence interval (CI) [11.0%, 27.1%], while the mean preoperative PJI risk percentage was 54.19 ± 16.58%. When stratified by revision indication, infection rates were 21.2% (14/66), 95% CI [13.1%, 32.5%] in septic revisions and 5.3% (1/19), 95% CI [0.9%, 24.6%] in aseptic revisions, with corresponding preoperative PJI risk values of 54.34 ± 16.95% and 53.65 ± 15.62%. Among the 15 patients who developed postoperative infections, the most common organisms were *Staphylococcus aureus* (*n* = 5) and *Staphylococcus epidermidis* (*n* = 5). Kidney complications occurred in 5.9% (5/85), 95% CI [0.8%, 11.0%], and wound complications in 21.2% (18/85), 95% CI [12.3%, 30.0%]. Implant removal or component exchange was required in 7.1% (6/85), 95% CI [1.5%, 12.6%].

**Conclusions:**

DABC (G + V) demonstrated favourable infection control in septic RTKA and effective infection prevention in aseptic RTKA, with a low complication rate and good overall safety.

**Level of Evidence:**

Level III.

**Abbreviations**
AKI: acute kidney injury
ALBC: antibiotic‐loaded bone cement
DABC: dual‐antibiotic bone cement
G+: Gram‐positive bacterial
IQR: interquartile range
MDT: multidisciplinary team
MRSA: methicillin‐resistant *Staphylococcus aureus*
PBC: plain bone cement
PJI: periprosthetic joint infection
RTKA: revision total knee arthroplasty
SABC: single‐antibiotic bone cement
TKA: total knee arthroplasty

## INTRODUCTION

In recent years, the increasing number of total knee arthroplasty (TKA) has been accompanied by a steady rise in associated revision procedures [37]. Considering that the postoperative infection rate of revision TKA (RTKA) is higher than that of primary TKA [16, 34], numerous strategies have been developed in recent years to prevent this devastating complication [2, 3, 28, 31, 39]. Among these, antibiotic‐loaded bone cement (ALBC) has played a considerable role in both the prevention and the management of periprosthetic joint infections (PJI). However, the use of ALBC in joint arthroplasty remains a topic of debate. While two studies found a reduced revision risk when using ALBC compared to plain bone cement (PBC) in primary TKA [19, 25], several contrasted these findings by finding non‐inferior results when using PBC compared to ALBC [13, 26].

Meanwhile, substantial research has been conducted on various aspects of ALBC, including its composition, material types, biological properties, types of incorporated antibiotics and antibiotic elution performance [1, 17, 18, 32]. The most commonly used ALBC typically incorporates a single antibiotic, such as gentamicin, clindamycin, vancomycin or tobramycin. An ideal antibiotic should meet the following criteria: broad antimicrobial spectrum, safety, thermal stability, water solubility and low allergenicity [17, 20, 35]. However, no single antibiotic currently satisfies all these requirements. To compensate for the limitations of single antibiotics and enhance the anti‐infective properties of bone cement, the combined use of two or even three complementary antibiotics within bone cement has become increasingly common in recent years [15, 21]. Nevertheless, the available evidence supporting dual‐antibiotic formulations remains limited and somewhat inconsistent, with most data derived from in vitro rather than clinical studies [14]. For example, the association of gentamicin and vancomycin is one of the combinations currently commercially available. Gentamicin, due to its broad‐spectrum activity, excellent thermal stability and favourable elution properties, has been used for decades in primary and revision hip and knee arthroplasty [23, 33]. Vancomycin, a glycopeptide antimicrobial agent, achieves bactericidal effects by inhibiting the synthesis of Gram‐positive bacterial (G+) cell walls, and it exhibits strong antibacterial activity against methicillin‐resistant *Staphylococcus aureus* (MRSA). The combination of these two agents provides complementary antimicrobial coverage, theoretically improving protection against both Gram‐negative and resistant Gram‐positive organisms. However, the real‐world performance of this DABC, especially in high‐risk revision TKA cases, has not yet been clearly established.

However, while promising from a theoretical point of view, clinical studies on dual‐antibiotic bone cement (DABC) are still scarce, with some in‐vitro studies demonstrating superior antimicrobial properties and better inhibition of biofilm formation compared to single‐antibiotic bone cement (SABC) [4, 6, 38]. This persistent uncertainty supports the need for further investigation into whether the gentamicin–vancomycin combination provides measurable clinical advantages in preventing and managing PJI in complex or high‐risk revision TKA settings.

The aim of this study was to evaluate whether DABC plays a role in both septic and aseptic RTKA, assessing its curative and preventive effects, respectively, after a minimum follow‐up of 2 years. It was further hypothesized that the application of DABC is safe for use in both septic and aseptic RTKA.

## METHODS

### Patients

**Figure 1:**
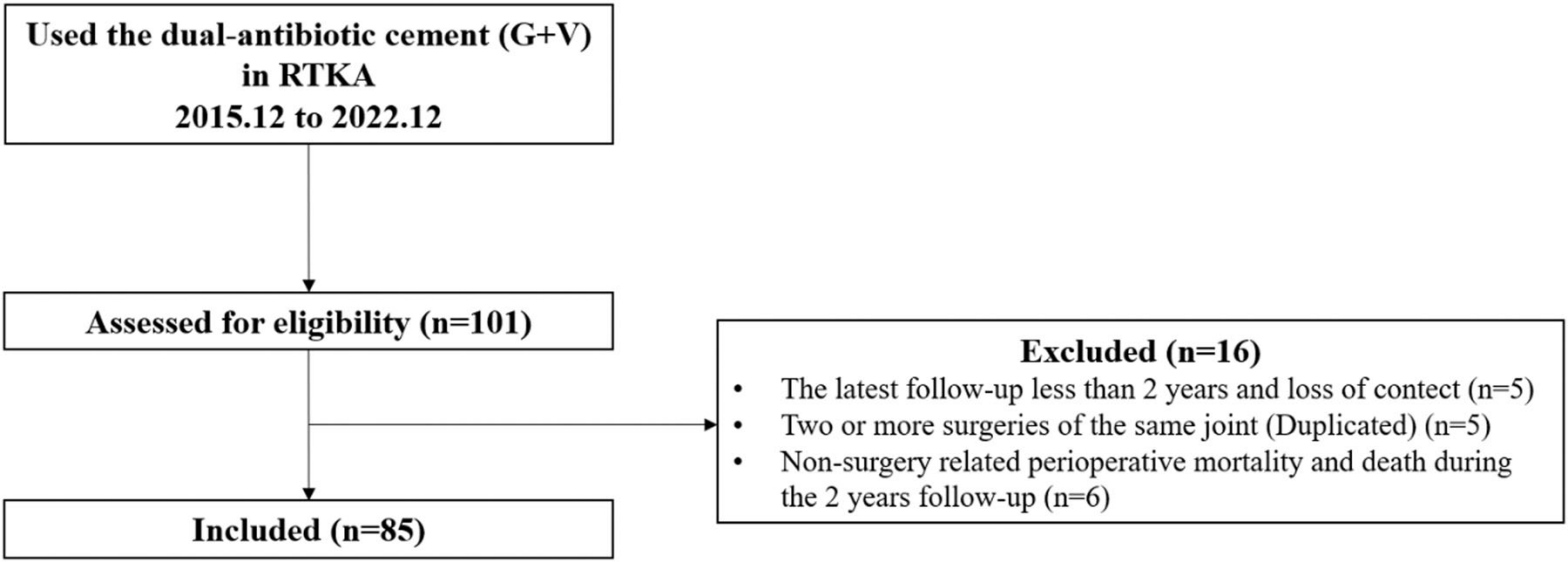
Flowchart visualizing the selection process of the eligible patients in the current study. G, gentamicin; RTKA, revision total knee arthroplasty; V, vancomycin.

From December 2015 to December 2022, 101 RTKA using DABC (gentamicin and vancomycin) were prospectively enroled into an institutional database at a referral centre for bone and joint infection (Croix Rousse Hospital, Hospices Civils de Lyon, France; Figure 1). The inclusion criteria were as follows:

(1) Cases involving RTKA where gentamicin–vancomycin cement was used for cementation, either for PJI prevention or PJI management: In high‐risk patients receiving RKTA for aseptic indications (e.g., those with obesity, malnutrition, diabetes, smoking history, multiple previous surgeries or prior infections at the same joint, based on a PJI score exceeding 150, according to Tan et al. [40]), DABC was used prophylactically for cementation of the components to reduce postoperative infection risk. In PJI cases, it was utilized both for cement spacers in case of two‐stage procedures when applicable and for cementing the components at the time of reimplantation.
(2) All patients had at least 2 years of postoperative follow‐up.

**Figure 2:**
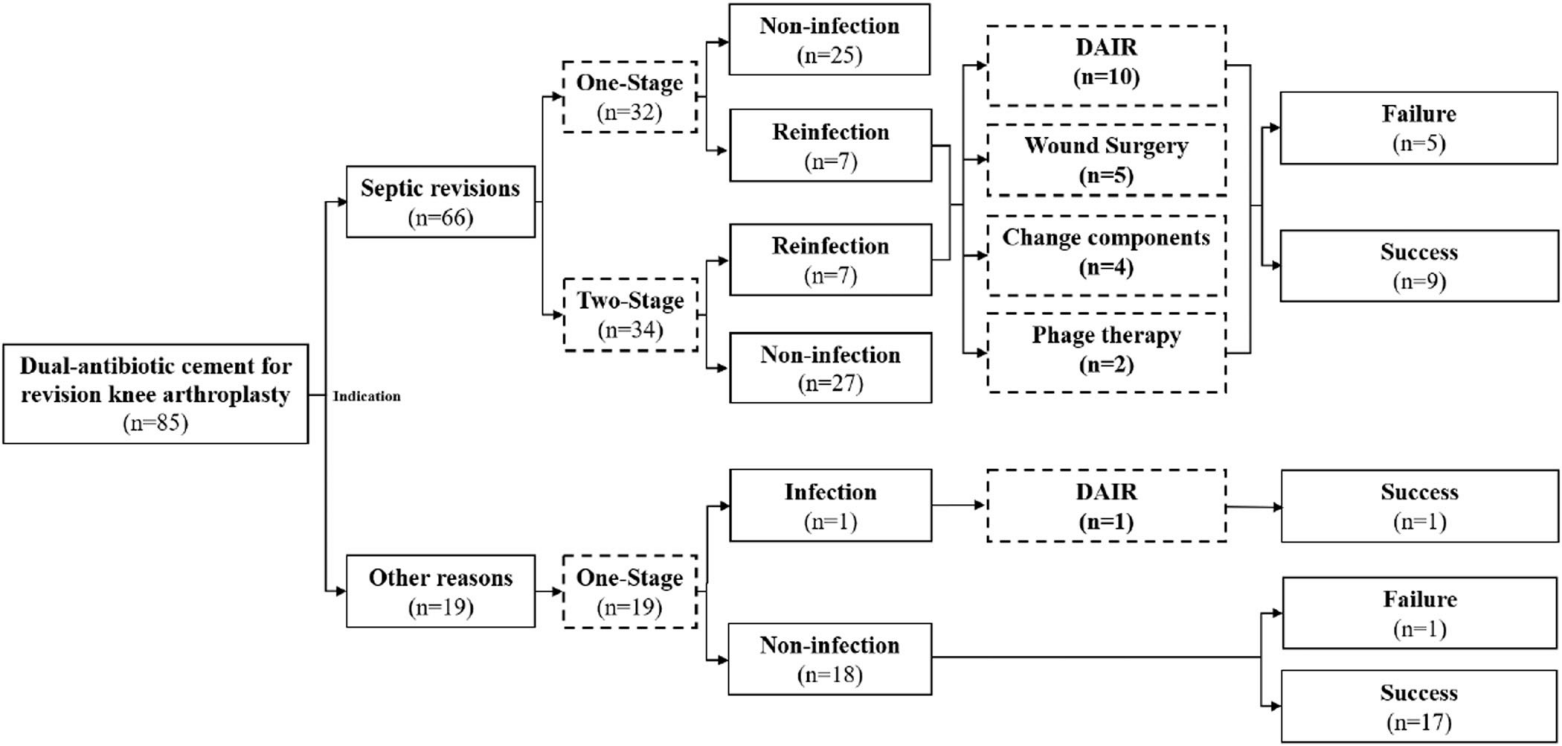
The graphical outcome and surgical treatment strategies. DAIR, debridement, antibiotics and implant retention.

Informed consent was obtained from patients and their families. A total of 85 patients were included in the final analysis with a mean follow‐up of 35.3 months (range: 24–84), of which 66 patients (77.6%) underwent RTKA due to PJI (Figure 2). Although the study period covered 7 years, the final cohort consisted of 85 patients because DABC was reserved exclusively for very high‐risk cases according to strict selection criteria (active PJI or a PJI Risk Score exceeding 150). Consequently, the sample size reflects the targeted use of DABC in this selected population rather than the overall RTKA volume of the centre. As two groups were defined in the inclusion criteria, patients were categorized based on the indications for revision surgery into infection‐related and non‐infection‐related revision groups for comparative analysis. Most of the indicators have no differences in preoperative demographics between the high‐risk patients receiving prophylactic DABC and those receiving DABC in case of PJI (Table 1).

**Table 1:** Summary of all cases' preoperative surgical characteristics (septic versus non‐septic revision).

Variable	Patients (*N* = 85)	Septic revision (*N* = 66)	Other reasons (*N* = 19)	*p* value	Effect size^a^
Age (years), means ± SD	68.75 ± 9.88	69.23 ± 9.66	67.11 ± 10.70	0.412	0.214 [−0.297, 0.725]
Gender, *n* (%)				0.204	0.138
Male	52 (61.2)	38 (57.6)	14 (73.7)		
Female	33 (38.8)	28 (42.4)	5 (26.3)		
BMI, means ± SD	30.44 ± 6.08	30.03 ± 6.50	31.88 ± 4.16	0.143	−0.306 [−0.817, 0.207]
ASA classification, *n* (%)				0.195	0.227
I	4 (4.7)	3 (4.5)	1 (5.3)		
II	33 (38.8)	22 (33.3)	11 (57.9)		
III	45 (52.9)	38 (57.6)	7 (15.6)		
IV	3 (3.5)	3 (4.5)	0 (0)		
Allergy, *n* (%)	43 (50.6)	33 (50.0)	10 (52.6)	0.840	0.022
Tabacco use, *n* (%)	9 (10.6)	8 (12.1)	1 (5.3)	0.665	0.093
Wound complication, *n* (%)	25 (29.4)	23 (34.8)	2 (10.5)	0.040	0.222
Kidney disorder preoperative, *n* (%)	11 (12.9)	9 (13.6)	2 (10.5)	1.000	0.039
No. of surgery before, means ± SD	4.15 ± 2.03	4.09 ± 1.85	4.37 ± 2.63	0.603	−0.136 [−0.646, 0.375]
Category of prosthesis, *n* (%)				0.880	0.088
1. Constrained Condylar Knee	26 (30.6)	19 (28.8)	7 (36.8)		
2. Rotating Hinge Knee	25 (29.4)	20 (30.3)	5 (26.3)		
3. Limb Preservation System	27 (31.8)	21 (31.8)	6 (31.6)		
4. Arthrodesis Implant	7 (8.2)	6 (9.1)	1 (14.3)		

Abbreviations: ASA, American Society of Anesthesiologists; BMI, body mass index; CI, confidence interval; SD, standard deviation.

^a^ Effect size: Cohen's *d* for continuous variables (with 95% CI), and Cramer's *V* for categorical variables (CI not reported).

### Surgical technique and follow‐up protocol

**Figure 3:**
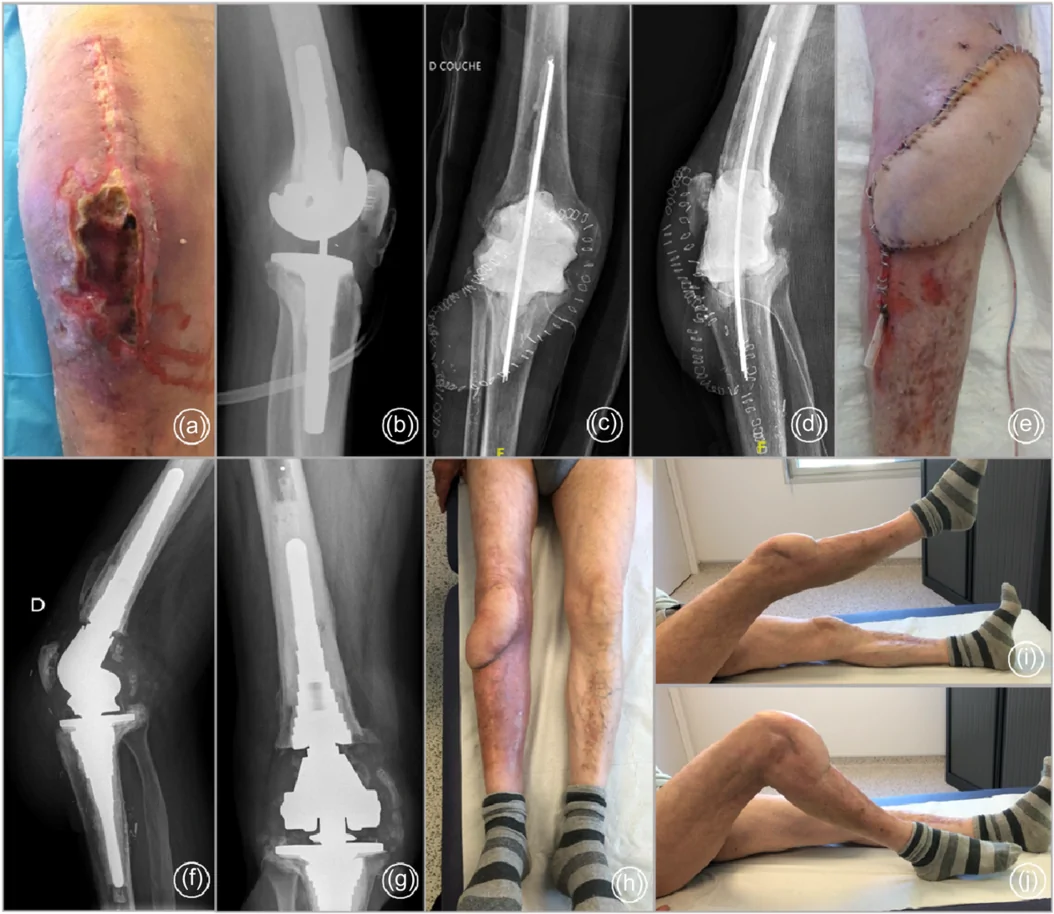
Example of the cases with dual‐antibiotic bone cement. This case is a male patient due to PJI and presented with redness, swelling, pain in the right knee, exhibited wound healing difficulties, sinus tract formation and persistent bone exposure (a). Preoperative X‐ray imaging revealed radiolucent lines around the prosthesis (b). After multidisciplinary discussion, a two‐stage RTKA was performed. The first stage involved debridement, removal of infected soft tissue and installation of the Copal (G + V) spacer (c, d), followed by flap coverage and transplantation (e). Once the infection was controlled, the second stage consisted of prosthesis reimplantation (f, g). Postoperatively, the patient received regular antibiotic therapy and demonstrated good recovery (h, i, j).

From Figure 3, all surgeries were performed by the senior author. In cases of PJI, the surgical protocol (one‐ or two‐stage) and postoperative antibiotic regimen were determined during a multidisciplinary team (MDT) meeting. In total, within this septic RTKA group, 32 cases were treated with a one‐stage RTKA, while 34 cases underwent a two‐stage procedure.

The one‐stage strategy was indicated when there was no evidence of systemic sepsis, no requirement for additional soft‐tissue coverage, no severe bone loss necessitating the use of a distal femoral or proximal tibial replacement, and no infection with a multi‐resistant organism [5]. The two‐stage strategy was adopted whenever any of these contraindications to the one‐stage approach were present.

The DABC used in this study is COPAL® G + V bone cement (Heraeus‐Medical GmbH), which is based on PALACOS® bone cement (Heraeus‐Medical GmbH) and loaded with approximately 0.86 g of gentamicin sulphate (= 0.5 g of active gentamicin) and 2.15 g of vancomycin hydrochloride (= 2 g of active vancomycin). All implants were cemented using DABC for both femur and tibia. All patients underwent the same fast‐track rehabilitation protocol and were followed up at 2 weeks, 6 weeks, 6 months, 1 year postoperatively and yearly afterwards. In cases of suspected infection postoperatively, its management was discussed with the assistance of the MDT.

### Outcomes

#### Primary outcome

The primary outcome of this study was the infection rate, including reinfection and new infection (as the primary endpoint), following revision surgeries using DABC. The infection rate was based on the presence of PJI, according to the International Consensus Meeting 2013 criteria [29] and its 2018 update [30]. Any of the following factors made the presence of any PJI likely as well: (1) wound healing issues with (recurrence of) infection, (2) occurrence of PJI‐suspected mortality and (3) necessity for suppressive antibiotic therapy. We defined treatment failure (as the secondary endpoint) as recurrent infection after the index surgery (using Copal G + V), with persistence of infection despite repeat salvage therapy or the need for amputation. The infection rate and failure rate were analysed separately for the septic and aseptic RTKA groups.

#### Secondary outcomes

(1) PJI risk was calculated for each patient according to the PJI risk calculator published by Tan et al. [40]. For both the aseptic and septic RTKA groups, the postoperative PJI rate was compared to the risk calculated with the PJI risk calculator.
(2) A distinction was made between recurrent or new infections in the septic TKA cohort, defined as (1) recurrent cases caused by strains identical in species and type to the microorganisms cultured intraoperatively; (2) infections caused by new pathogens distinct from the original isolates occurring during or after treatment. For cases with reoccurring PJI during postoperative follow‐up, bacteriological data were collected to evaluate microbiological and sensitivity to G + V differences before and after the surgery.
(3) Safety of this DABC was analysed by evaluating kidney disorders (acute kidney injury, AKI), wound complications (including fistula and skin necrosis), mechanical complications (including loosening, dislocation, periprosthetic fractures, instability) and ultimately implant removal due to loosening.

### Statistical analysis

Statistical analyses were performed using SPSS 27.0 (IBM) software. In case parametric conditions were met for continuous variables, comparisons between two groups were performed using the *t* test and presented as mean ± standard deviation. In case parametric conditions were not met, the Mann–Whitney *U* test was used, and presented as median (interquartile range, IQR). Categorical variables were expressed as counts (*n*) and percentages (%), and comparisons of distributions were performed using the *χ* ^2^ test or Fisher's exact test. To better quantify the magnitude of differences between groups, effect sizes were calculated for the comparative indicators and are reported in the tables. For continuous variables, Cohen's *d* was computed for parametric tests and rank‐biserial correlation (*r*) for non‐parametric tests. For categorical variables, Cramer's *V* was reported. A *p* value of <0.05 was considered statistically significant.

## RESULTS

### Preventive and curative effects

Postoperative PJI occurred in 21.2% (14/66) in the septic RTKA group (21.9% for one‐stage procedures; 20.6% for two‐stage procedures), whereas only 5.3% (1/19) in the aseptic revision group developed postoperative PJI (Figure 2). At final follow‐up, treatment failure was observed in 7.6% (5/66) in the septic revision group and 5.3% (1/19) in the aseptic revision group (Figure 2). In the septic RTKA group, the mean PJI Risk Score was 54.34 ± 16.95%, and the observed infection rate was 21.2% (Table 2). Among the 19 cases of the aseptic RTKA cohort, the mean PJI Risk Score was 53.65 ± 15.62%, with an observed infection rate of 5.3%. When grouped by postoperative infection (the primary endpoint) rather than surgical indication, there were no differences in demographics or surgical details between patients who were infected or those who remained infection‐free (Table 3).

**Table 2:** Preoperative risk and postoperative actual infection rate of patients.

Variable	Patients (*N* = 85)	Septic revision (*N* = 66)	Other reasons (*N* = 19)
PJI Risk Score, means ± SD	223.79 ± 29.56	224.12 ± 30.37	222.63 ± 27.32
PJI risk percentage (%), means ± SD	54.19 ± 16.58	54.34 ± 16.95	53.65 ± 15.62
Postoperative infection rate (%, 95% CI)	17.6 [11.0, 27.1]	21.2 [13.1, 32.5]	5.3 [0.9, 24.6]
Failure rate (%, 95% CI)	7.1 [3.3, 14.6]	7.6 [3.3, 16.5]	5.3 [0.9, 24.6]

Abbreviations: CI, confidence interval; PJI, periprosthetic joint infection; SD, standard deviation.

**Table 3:** Summary of all cases' preoperative surgical characteristics (non‐septic complication versus septic complication).

Variable	Patients (*N* = 85)	No septic complication (*N* = 70)	Reinfection/New infection (*N* = 15)	*p* value	Effect size^a^
Age (years), means ± SD	68.75 ± 9.88	68.53 ± 9.17	69.80 ± 13.02	0.654	0.128 [−0.430, 0.686]
Gender, *n* (%)				0.631	0.052
Male	52 (61.2)	42 (60.0)	10 (66.7)		
Female	33 (38.8)	28 (40.0)	5 (33.3)		
BMI, means ± SD	30.44 ± 6.08	30.08 ± 5.69	32.14 ± 7.65	0.338	0.339 [−0.222, 0.898]
ASA classification, *n* (%)				0.398	0.206
I	4 (4.7)	4 (5.7)	0 (0.0)		
II	33 (38.8)	29 (41.4)	4 (26.7)		
III	45 (52.9)	34 (48.6)	11 (73.3)		
IV	3 (3.5)	3 (4.3)	0 (0)		
Allergy, *n* (%)	43 (50.6)	35 (50.0)	8 (53.3)	0.815	0.025
Tabacco use, *n* (%)	9 (10.6)	6 (8.6)	3 (20.0)	0.399	0.142
Wound complications, *n* (%)	25 (29.4)	20 (28.6)	5 (33.3)	0.956	0.040
Kidney disorder before surgery, *n* (%)	11 (12.9)	9 (12.9)	2 (13.3)	1.000	0.005
No. of surgery before, means ± SD	4.15 ± 2.03	3.99 ± 1.89	4.93 ± 2.52	0.102	0.471 [−0.093, 1.032]
Indication of revision, *n* (%)				0.602	0.177
Septic revisions	66 (77.6)	52 (74.3)	14 (93.3)		
Aseptic loosening	16 (18.8)	15 (21.4)	1 (6.7)		
Stiffness	1 (1.2)	1 (1.4)	0 (0.0)		
Other reasons	2 (2.4)	2 (2.9)	0 (0.0)		
Stage of surgery, *n* (%)				0.561	0.063
I	51 (60.0)	43 (61.4)	8 (53.3)		
II	34 (40.0)	27 (38.6)	7 (46.7)		
Use the muscular flap, *n* (%)	9 (10.6)	6 (8.6)	3 (20.0)	0.399	0.142
Category of prosthesis, *n* (%)				0.348	0.183
1. Constrained Condylar Knee	26 (30.6)	24 (34.3)	2 (13.3)		
2. Rotating Hinge Knee	25 (29.4)	20 (28.6)	5 (33.3)		
3. Limb Preservation System	27 (31.8)	21 (30.0)	6 (40.0)		
4. Arthrodesis Implant	7 (8.2)	5 (7.1)	2 (13.3)		

Abbreviations: ASA, American Society of Anesthesiologists; BMI, body mass index; CI, confidence interval; SD, standard deviation.

^a^ Effect size: Cohen's *d* for continuous variables (with 95% CI) and Cramer's *V* for categorical variables (CI not reported).

In the septic RTKA group, recurrent PJI with the same microorganism as the initial infection was identified in only 3 out of 15 cases (Table 4). The sensitivity rate to gentamicin increased from 46.7% (7/15) preoperatively to 86.7% (13/15) postoperatively, while the sensitivity rate to vancomycin decreased slightly from 73.3% (11/15) preoperatively to 66.7% (10/15) postoperatively.

**Table 4:** Microbiology results and information on the infection cases.

No.	Patients characteristic			Indication	Pre‐operative microbiology			Surgical strategy	Post‐operative microbiology			Intervention	Outcome
	Gender	Age	Side		Isolated microorganism	Sensitivity to gentamycin	Sensitivity to vancomycin		Isolated microorganism	Sensibility to gentamycin	Sensibility to vancomycin		
1	Male	71	Right	Septic revision	*Staphylococcus epidermidis*	R	S	Two‐stage	*Streptococcus agalactiae*/*Staphylococcus aureus*	S	S	DAIR	Success
2	Female	84	Right	Septic revision	*S. epidermidis*	R	S	One‐stage	*S. aureus*/*Actinomyces odontolyticus*/*Streptococcus mitis*/*Streptococcus oralis*	S	S	DAIR	Failure
3	Male	73	Left	Septic revision	*S. aureus*	S	S	Two‐stage	*S. aureus*	S	S	DAIR	Failure
4	Male	64	Left	Septic revision	*S. aureus*	S	S	Two‐stage	*S. aureus*	S	S	DAIR + change tibial prosthesis	Success
5	Male	49	Right	Septic revision	*Enterococcus faecium*	R	S	Two‐stage	*Citrobacter koseri*/*Cutibacterium acnes*	S	S	DAIR + change polyethylene	Success
6	Male	81	Left	Septic revision	*Pseudomonas aeruginosa*	ND	ND	One‐stage	*S. aureus*/*Staphylococcus epidermidis*	S	S	DAIR	Success
7	Male	70	Left	Septic revision	*S. aureus*	S	S	One‐stage	*Klebsiella oxytoca*	S	ND	Amputation	Failure
8	Female	66	Left	Septic revision	*S. aureus*	S	S	Two‐stage	*Streptococcus dysgalactiae*	ND	S	DAIR + remove cortical screws; scar revision surgery	Success
9	Male	37	Left	Septic revision	*S. aureus*	S	S	Two‐stage	*Escherichia coli*	S	ND	DAIR + VAC; amputation	Failure
10	Female	71	Right	Septic revision	*Corynebacterium*	ND	ND	One‐stage	*Morganella morganii*/*Achromobacter xylosoxidans*/*Achromobacter denitrifricans*	R	ND	DAIR + flap transplantation	Success
11	Female	88	Right	Septic revision	*P. aeruginosa*	ND	ND	One‐stage	*Enterobacter cloacae*	S	ND	DAIR + scar revision surgery (phage)	Success
12	Male	65	Left	Septic revision	*S. epidermis*	S	S	One‐stage	*S. epidermis*	S	S	DAIR + change polyethylene (phage)	Success
13	Male	75	Right	Septic revision	*S. epidermis*	S	S	One‐stage	*S. dysgalactiae*	S	S	DAIR + change polyethylene	Success
14	Male	78	Left	Aseptic loosening	None	ND	ND	One‐stage	*E. coli*	S	ND	DAIR + VAC; flap transplantation	Success
15	Female	75	Left	Septic revision	*S. epidermidis*	R	S	Two‐stage	*Staphylocoque hominis*	S	S	Amputation	Failure

Abbreviations: DAIR, debridement, antibiotics and implant retention; ND, not done; R, resistant; S, sensitive; VAC, vacuum‐assisted closure.

### DABC safety

Postoperative follow‐up indicators revealed that 5.9% (5/85), 95% CI [0.8%, 11.0%] of patients developed kidney complications, 28.2% (24/85), 95% CI [18.5%, 38.0%] experienced mechanical complications, 21.2% (18/85), 95% CI [12.3%, 30.0%] had dermatological complications and 7.1% (6/85), 95% CI [1.5%, 12.6%] required implant removal (Table 5). When comparing patients undergoing septic RTKA and aseptic RTKA, no statistically significant differences were observed in kidney complications, mechanical implant failure, wound complications or implant removal rates.

**Table 5:** Summary of all patients' follow‐up and complications.

Variable	Patient (*N* = 85)	Septic revision (*N* = 66)	Other reasons (*N* = 19)	*p* value	Effect size^a^
Follow‐up (months, means ± SD)	35.32 ± 12.62	37.33 ± 13.57	28.32 ± 3.42	<0.001	0.744 [0.220, 1.265]
Kidney disorder after surgery (%, 95% CI)	5.9 [0.8, 11.0]	6.1 [0.1, 12.0]	5.3 [−5.8, 16.3]	1.000	0.014
Mechanical complication (%, 95% CI)	28.2 [18.5, 38.0]	27.3 [16.2, 38.3]	31.6 [8.6, 54.6]	0.713	0.040
Wound complication (%, 95% CI)	21.2 [12.3, 30.0]	22.7 [12.3, 33.1]	15.8 [−2.3, 33.8]	0.739	0.071
Implant removal (%, 95% CI)	7.1 [1.5, 12.6]	7.6 [1.0, 14.1]	5.3 [5.8, 16.3]	1.000	0.038

Abbreviations: CI, confidence interval; SD, standard deviation.

^a^ Effect size: Cohen's *d* for continuous variables (with 95% CI) and Cramer's *V* for categorical variables (CI not reported).

## DISCUSSION

To the best of our knowledge, this series represents the largest cohort of patients receiving DABC (gentamicin + vancomycin) during RTKA associated with at least 2 years of follow‐up. The main finding of the present study is that even in complex cases with high PJI Risk Scores, the use of DABC was associated with acceptable postoperative PJI rates in the challenging context of revision surgery. Using DABC in the septic RTKA, the reinfection rate was 21%, which was less than half of their calculated PJI Risk Score. Similarly, the infection rate was 5% in the high‐risk aseptic RTKA group compared to 54% according to the PJI Risk Score.

DABC with gentamicin and vancomycin (COPAL®) appears to have both a curative effect in septic RTKA and a preventive effect in aseptic RTKA. The infection rate observed in this study (21%) was comparable to other published series investigating DABC in septic RTKA, such as Yang et al. (18%) [42] and Corro et al. (23%) [10]. Although these results fall within the range reported in the literature (up to 30%) [24], the rate cannot be characterized as low but rather consistent with previous evidence, considering the complexity of this cohort. Furthermore, our study had a considerably larger sample size than these previous studies, which analysed 31 and 47 knee joints, respectively, although their cohorts included only two‐stage revisions, while ours also encompassed one‐stage procedures selected by the MDT.

When comparing our findings to studies on SABC, Wan et al. reported a 91% infection eradication rate (9/33) in their septic TKA cohort undergoing two‐stage revision after a 2‐year follow‐up, which appears superior to the results of this study [41]. However, a meta‐analysis found that ALBC with erythromycin and colistin or vancomycin did not significantly reduce PJI rates compared to PBC, underscoring the ongoing uncertainty regarding optimal antibiotic combinations [13]. Therefore, direct comparisons across studies should be interpreted cautiously, as patient complexity and procedural heterogeneity may strongly influence outcomes.

On the other hand, our results also suggest a preventive potential of DABC with gentamicin and vancomycin, with an infection rate of 5% in high‐risk patients undergoing aseptic RTKA, which was eight‐fold lower than the calculated mean PJI risk (54%). According to current literature, the PJI risk in aseptic RTKA is between 3% and 9%, which is higher than in the primary TKA setting [8]. Meanwhile, the infection rate of aseptic RTKA in this study was higher than in the cohort by Blersch et al. (1.2%), who analysed 403 cases of aseptic RTKA using DABC (gentamicin + clindamycin) [6]. Any difference between our study and the current literature might be due to the difference in patient population, as our cohort included patients considered high‐risk to develop PJI postoperatively, whereas no specific risk stratification was made in the study by Blersch et al. [6].

This study also analysed the microbiological profiles of patients with recurrent infections. Among those who experienced postoperative infections, the most common pathogens identified preoperatively were *S. aureus* and *Staphylococcus epidermidis*. Previous studies have reported similar findings, Corona et al. identified coagulase‐negative *S. aureus* as the most frequent microorganism in intraoperative cultures during the first stage of revision surgery [9]; Cabo et al. found that among 44 infected prostheses, *S. epidermidis* was the most common pathogen (18 cases), followed by *S. aureus* (8 cases) [7]; Sebastian et al., in their study of 286 revision TKA cases, reported *S. aureus* as the most commonly isolated pathogen [36]. Overall, the microbiological evidence observed in this study aligns with previous reports.

Regarding antibiotic susceptibility test results, in patients with recurrent infections, the sensitivity rates to gentamicin increased from 46.7% preoperatively to 86.7% postoperatively, while for vancomycin, they decreased from 80% to 66.7%, respectively. This alteration suggests that the introduction of DABC (gentamicin + vancomycin) may have selectively reduced the proportion of gentamicin‐resistant strains, improving its local effectiveness, while the slight decline in vancomycin sensitivity likely reflects the persistence of some vancomycin‐resistant organisms rather than the development of new resistance. Overall, these findings indicate that the use of DABC does not promote a clinically relevant increase in bacterial resistance.

Postoperative wound and kidney complications occurred in 21% and 6% of cases, respectively. These rates were within the range reported in previous series. Koressel et al. followed 186 RTKA for 2 years and found that seven patients (2.4%) required reoperation septic revision TKA due to superficial wound‐related issues, while excluding deep infections [22]. The observed difference may be attributed to our broader definition of wound complications, which includes both superficial and deep infections. Regarding kidney complications, the local antimicrobial release from bone cement differs significantly from systemic administration. Although Courtney et al. identified the addition of vancomycin to cefazolin as an independent risk factor for AKI following primary total joint arthroplasty [11], the 6% incidence of kidney complications observed here was lower than in similar studies (Edelstein et al. 27% [12]; Li et al. 12.1% [27]), suggesting that nephrotoxicity related to DABC is uncommon. Since kidney function is influenced by multiple perioperative factors, including surgery, anaesthesia, intravenous medication and nutritional status, a direct causal relationship between DABC use and wound or kidney complications cannot be established.

This study has several limitations. Firstly, this study was monocentric and retrospective, potentially limiting the generalizability of our results. Moreover, the lack of a control group makes it difficult to directly compare the results with alternative treatments. As the study was conducted at a high‐volume centre specialized in revision TKA and osteoarticular infections, the results should be interpreted in the light of more complex pathology on one hand, and a team highly specialized in infections on the other. Second, this study evaluated only one type of DABC longitudinally, necessitating caution against overinterpretation and making it challenging to determine whether this formulation is superior to other types. Nevertheless, the selection of antibiotics in bone cement in this study was based not only on microbiological evidence but also on patients' infection history and risk profile. Third, for the cases of aseptic knee revision, the use of this DABC was not the first‐line choice but was considered after thorough evaluation of patients' medical history and infection risk, thereby limiting the number of patients included in this group. The PJI risk calculator was chosen because of its large validation cohort (27,717 patients) and inclusion of 17 weighted risk factors, providing a comprehensive infection risk profile (mean 54%). While this score cannot be directly compared with the observed infection rate, it offers an overall estimate of postoperative reinfection risk in this complex population. Future research should refine such tools to better predict recurrence risk in patients already affected by PJI.

Finally, despite a minimum 2‐year follow‐up, residual confounding factors and limited sample size remain potential sources of bias. Future studies with larger multicenter cohorts and longer follow‐up are needed to validate these findings and further clarify the role of DABC in high‐risk revision TKA.

## CONCLUSIONS

The use of DABC in RTKA for high‐risk patients demonstrated both therapeutic and preventive effects against infection, with 21% of post‐operative infections in septic RTKA and 5% in aseptic RTKA, with follow‐up of at least 2 years of follow‐up. This satisfying rate of success is interesting as these are complex cases, sometimes requiring skin coverage using flap on multi‐operated knees. It also exhibited a favourable safety profile with minimal complications directly accountable to DABC.

## AUTHOR CONTRIBUTIONS

**Shengdong Yang**: Conceptualization; methodology; data curation; writing original draft. **Nicolas Cance**: Data curation and review; editing original draft. **Cécile Batailler**: Supervision; reviewing and editing. **Tristan Ferry**: Data curation; reviewing and editing. **Pierre Longlune**: Data inspection. **Luca Andriollo**: Reviewing and editing. **Hannes Vermue**: Reviewing and editing. **Sébastien Lustig**: Conceptualization; supervision, validation, reviewing and editing. The first and last authors contributed to the study's conception and design. Material preparation, data collection, analysis and the draft of the manuscript were performed by the first author. All authors commented on previous versions of the manuscript and approved the final manuscript.

## CONFLICT OF INTEREST STATEMENT

The authors declare that this research was conducted in the absence of any financial interests or personal relationships that could be construed as a potential conflict of interest. Cécile Batailler is a consultant for Stryker. Sébastien Lustig is a consultant for Stryker, Smith and Nephew, Heraeus and Depuy Synthes; institutional research support to Lepine and Amplitude; editorial board for *Journal of Bone and Joint Surgery* (*Am*). The remaining authors declare no conflict of interest.

## ETHICS STATEMENT

All procedures were performed in accordance with the ethical standards of the institutional and/or national research committee, the 1964 Helsinki Declaration and its later amendments or comparable ethical standards. Data collection and analysis were carried out in accordance with MR004 Reference Methodology from the Commission Nationale de l′Informatique et des Libertés (Ref. 2226075) obtained on 19 April 2022. The study was registered and filed on the Health Data Hub website. Written informed consent was obtained from all patients and/or families.

## Data Availability

The datasets used and analysed during the current study are available from the corresponding author on reasonable request, subject to approval by the ethics committees of Croix‐Rousse Hospital.
